# Evaluation of the Reddesa Chart, a New Red Desaturation Testing Method, for Optic Neuritis Screening and Grading in Clinical Routine

**DOI:** 10.3389/fneur.2022.898064

**Published:** 2022-07-07

**Authors:** Dominik Bruegger, Anna-Lucia Koth, Muriel Dysli, David Goldblum, Mathias Abegg, Markus Tschopp, Christoph Tappeiner

**Affiliations:** ^1^Department of Ophthalmology, Inselspital, Bern University Hospital, Bern, Switzerland; ^2^Augenpraxis Untertor, Winterthur, Switzerland; ^3^Department of Ophthalmology, Cantonal Hospital Aarau, Aarau, Switzerland; ^4^Pallas Kliniken, Olten, Switzerland; ^5^Department of Ophthalmology, Pallas Klinik, Olten, Switzerland; ^6^Department of Ophthalmology, University Hospital Essen, University Duisburg-Essen, Essen, Germany; ^7^University of Bern, Bern, Switzerland

**Keywords:** red desaturation, optic neuritis, screening, red cap test, Reddesa chart

## Abstract

**Background:**

Optic neuritis usually leads to reduced color sensitivity. Most often, the change of red color, the so-called red desaturation, is tested in clinical routine. The aim of this study was to test the feasibility of the Reddesa chart, a new red desaturation test based on polarization, as a screening method for optic neuropathy.

**Methods:**

A total of 20 patients with unilateral optic neuritis and 20 healthy controls were included in this prospective pilot study. Ophthalmological examination included assessment of best corrected visual acuity (BCVA), slit lamp examination, fundoscopy, testing of relative afferent pupillary defect (RAPD) and red desaturation with the red cup test and the Reddesa chart.

**Results:**

The mean BCVA in the optic neuritis group was 0.76 ± 0.36 in the affected eye (95% of eyes with RAPD, 75% of eyes with difference in the Reddesa test) and 1.28 ± 0.24 in the healthy eye, whereas in the control group, BCVA was 1.14 ± 0.11 in the right eye and 1.15 ± 0.14 in the left eye (none of the eyes with RAPD or abnormal Reddesa test). In our study, the Reddesa test showed a positive predictive value of 100% and a negative predictive value of 80% for detecting optic neuritis.

**Conclusion:**

The Reddesa chart allows to quantify red desaturation and has the potential to be implemented as a screening test in clinical routine.

## Introduction

Optic neuritis, an acute inflammation of the optic nerve, is associated with demyelinating diseases such as multiple sclerosis and other autoimmune disorders but may occur also due to infections (1–3). Incidence of optic neuritis of up to 6.4 per 100'000 has been reported (2, 4–6). Optic neuritis is associated with demyelinating disease and occurs more commonly in women with a mean age of 31.8 years (1). Impairment of color vision is typical in eyes with optic neuritis and appears disproportional to visual loss (7). The Optic Neuritis Study Group found an abnormal color vision in 94% of patients in the acute phase of optic neuritis disease, which persisted in 40% of them as residual color defects even after 6 months (1). Optic neuritis often leads to red–green deficiencies (8). However, a variety of other color deficiencies (e.g., blue–yellow and non-specified color defects) have been reported in these patients (7, 9).

In clinical routine, testing of red desaturation is commonly performed, especially in patients with acute vision loss of unknown etiology. Red objects presented to such patients are perceived as grayish due to the reduced red saturation compared to the other healthy eye. Often, the red cap of topical ocular medications is presented to the patient, hence the name “red cap test.” However, up to 25% of patients without apparent optic neuritis are false positive in the red cap test (10). The quantification of color defects, e.g., with neutral density filters, is not commonly done by practitioners.

We designed a new red desaturation testing chart, the Reddesa test, that allows the quantification of the red desaturation. In this pilot study, we evaluate the feasibility of using the Reddesa test in a clinical setting.

## Methods

**Figure 1:**
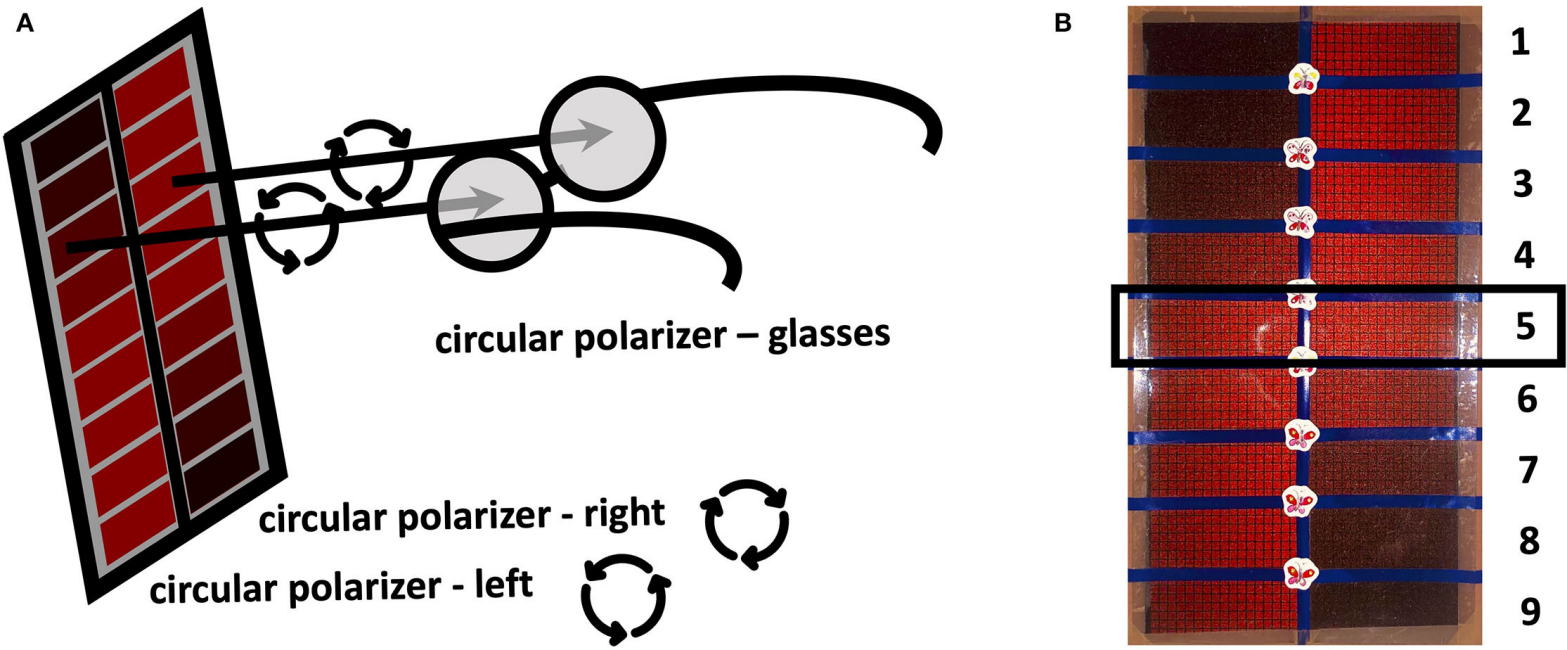
Schematic illustration of the Reddesa chart **(A)**. The patient is wearing polarization glasses and holds the Reddesa test at reading distance. He/she has to identify the field on the left and right columns that appear to have the same red saturation. Photograph of the Reddesa test chart **(B)**. The graduations of the red color change gradually in the fields of two rows next to each other. The graduation in the middle (number 5) denotes no difference in the red desaturation of both eyes.

In this prospective monocentric pilot trial, a total of 20 consecutive patients with unilateral optic neuritis and ≥18 years of age were included. Optic neuritis diagnosis was based on the medical history, clinical examination (e.g., the presence of a relative afferent pupillary defect, abnormal red cap test, etc.) and if needed on MRI confirmation. Exclusion criteria were bilateral optic neuritis and any other ocular, optic nerve, or central pathology. A cohort of 20 healthy probands was included as a control group. All study participants were examined by a board-certified ophthalmologist in a clinical examination including best-corrected visual acuity (BCVA) testing, slit lamp examination, and fundoscopy. Furthermore, a Lang stereo test and a red cap test were performed in all study participants. For the red cap test, we used the red cover of a Lang stereo test plate to have a standardized red object. For this test, the patients had to alternately close one eye and denote whether they perceived a difference in the color/saturation of the red color. Furthermore, we designed a new test, the so-called Reddesa test, for quantifying the red desaturation severity. The test consists of a chart template with 1–9 graduations of the red color on two columns next to each other (Figures 1A,B). The template was printed on an aluminum dibond plate. The different graduations of red were designed in Adobe® Photoshop, simulating different neutral density (ND) filters above the red color. We used the following ND filters: 0 (transmission 100%) for row 5; 0.15 (transmission 71%) left side row 4; 0.3 (transmission 50%) left side row 3; 0.45 (transmission 35%) left side row 2; 0.6 (transmission 25%) left side row 1; 0.15 (transmission 71%) right side row 6; 0.3 (transmission 50%) right side row 7; 0.45 (transmission 35%) left side row 8; 0.6 (transmission 25%) right side row 9. By adding a different circular polarizing filter on each side of the chart and wearing glasses with the corresponding polarizing filter, each eye can see one-half of the template (Figure 1A). The patient holds the template in his hands at reading distance. Due to the glasses with the polarizing filters, each eye sees only the corresponding row of 9 fields that change in the degree of red color saturation successively (Figure 1A). The patient designates the row where he/she perceives the red color as similar in both fields. Hence, the reduced saturation of the red print presented to the healthy eye matches the red desaturation of the pathological eye (to which the full color is presented). For example, in row 1, the left eye sees the left field of the panel with a transmission of 25% and the right eye sees the right field of the panel with a transmission of 100%; in row 9, the left eye sees the left field of the panel with a transmission of 100% and the right eye sees the right field of the panel with a transmission of 25%. Therefore, if a patient perceives the left and the right fields in row 1 as similar, a red desaturation of the right eye may be assumed, whereas if this occurs in row 9, a red desaturation of the left eye may be assumed. If there is no difference in red desaturation of both eyes, the patient is expected to choose the graduation in the two fields in the middle, i.e., field number 5 (Figure 1B). In our pilot study, the Reddesa test was performed one time at usual room brightness of 300–500 lux and one time in dim light of 10–50 lux.

All statistical evaluations were performed using Prism 5.0c (GraphPad Software, La Jolla, USA). Data are presented as means ± standard deviation (SD). Unpaired *t*-tests and Fisher's exact tests were used for group comparisons, as appropriate. The correlation between BCVA difference (between the right and left eye) to the Reddesa findings was assessed using Spearman-rho correlation analysis. *p*-Values < 0.05 were considered statistically significant.

The study has been approved by the responsible ethical board and was performed in accordance with the Declaration of Helsinki. All study participants have signed a written informed consent before inclusion into the study.

## Results

A total of 20 patients with optic neuritis (group 1; Table 1) and 20 healthy controls (group 2) were included in our study. In group 1, the mean age was 37.6 ± 13.6 years (65% women), whereas in group 2, it was 31.1 ± 6.8 years (80% women; *p* > 0.05; Table 2). The mean duration of symptoms in the neuritis group was 43.4 ± 50.8 days (range 0–151). The red cap test was abnormal in 19 out of 20 patients in the neuritis group on the affected eye, whereas no difference between both eyes was found in all subjects of the healthy control group. A total of 19 out of 20 patients in the neuritis group revealed a RAPD on the affected eye (none in the control group). In the neuritis group, the Reddesa test showed a difference between both eyes in 15 out of 20 patients (Table 1). On the other hand, in the healthy control group, the Reddesa test was normal in all subjects. No significant difference in the ratio of patients with an abnormal red cap test and an abnormal Reddesa test was found in the neuritis group and in the healthy control group, respectively (*p* > 0.05, each).

**Table 1:** Clinical characteristics of patients with optic neuritis (*N* = 20).

**Pat**	**Age**	**Sex**	**Duration of**	**Affected**	**RAPD**	**BCVA**	**BCVA**	**Lang stereo**	**Red cap**	**Reddesa**	**Reddesa**
**No**	**(y)**		**symptoms (d)**	**eye**		**RE**	**LE**	**test**	**test**	**300-500 lux**	**10-50 lux**
1	66	m	22	LE	LE	1.0	0.63	neg	LE minimal difference	8	7
2	19	f	55	RE	RE	1.25	1.6	neg	Normal	5	5
3	57	m	17	RE	RE	0.8	1.25	neg	RE slightly brighter	3	5
4	41	f	125	RE	RE	1.25	1.6	pos	RE darker	1	1
5	35	m	1	RE	RE	0.32	1.0	neg	RE minimal difference	3	3
6	47	m	151	LE	LE	1.0	0.5	pos	Minimal difference	7	8
7	39	f	22	RE	no	1.25	1.25	pos	Minimal difference	5	6
8	39	f	3	LE	LE	1.0	0.5	neg	Difference	9	7
9	54	f	26	RE	RE	1.25	1.25	pos	LE pale	5	5
10	41	m	63	RE	RE	1.0	1.6	neg	Minimal difference	1	1
11	22	f	1	RE	RE	1.0	1.25	pos	Grayish	3	3
12	47	f	75	RE	RE	1.0	1.6	pos	Minimal difference	5	5
13	20	f	6	RE	RE	0.5	1.25	pos	RE pale	4	3
14	40	m	1	LE	LE	1.0	0.2	neg	LE pale	9	9
15	52	f	114	RE	RE	0.32	1.6	pos	RE grayish	1	1
16	36	f	16	LE	LE	1.25	0.25	neg	LE minimal opaque	7	9
17	20	f	7	RE	RE	0.5	1.25	pos	RE pale	2	3
18	28	m	22	LE	LE	1.6	1.0	pos	LE pale	6	5
19	27	f	0	RE	RE	1.0	1.0	pos	RE pale	3	4
20	23	f	141	RE	RE	0.8	1.25	pos	RE pale	5	5

*BCVA, best-corrected visual acuity (decimal); RE, right eye; LE, left eye; RAPD, relative afferent pupillary deficit*.

**Table 2:** Descriptive statistics of patients with optic neuritis and healthy controls.

	**Neuritis patients (*N* = 20)**	**Healthy controls (*N* = 20)**	***p-*value**
Age (y), mean ± SD	37.7 ± 13.6	31.1 ± 6.8	>0.05
Females (%)	65%	80%	>0.05
BCVA better eye (logMar), mean ± SD	−0.10 ± 0.08	−0.06 ± 0.04	>0.05
BCVA worse eye (logMar), mean ± SD	0.18 ± 0.25	−0.06 ± 0.05	0.0002
Duration of symptoms (d), mean ± SD	43.4 ± 50.8	n.a.	n.a.
RAPD (%)	95%	0%	<0.0001
Abnormal red cap test (%)	95%	0%	<0.0001
Abnormal Reddesa test; room light (%)	75%	0%	<0.0001
Abnormal Reddesa test; dim light (%)	70%	0%	<0.0001

*BCVA, best-corrected visual acuity; SD, standard deviation; n.a., not applicable*.

**Figure 2:**
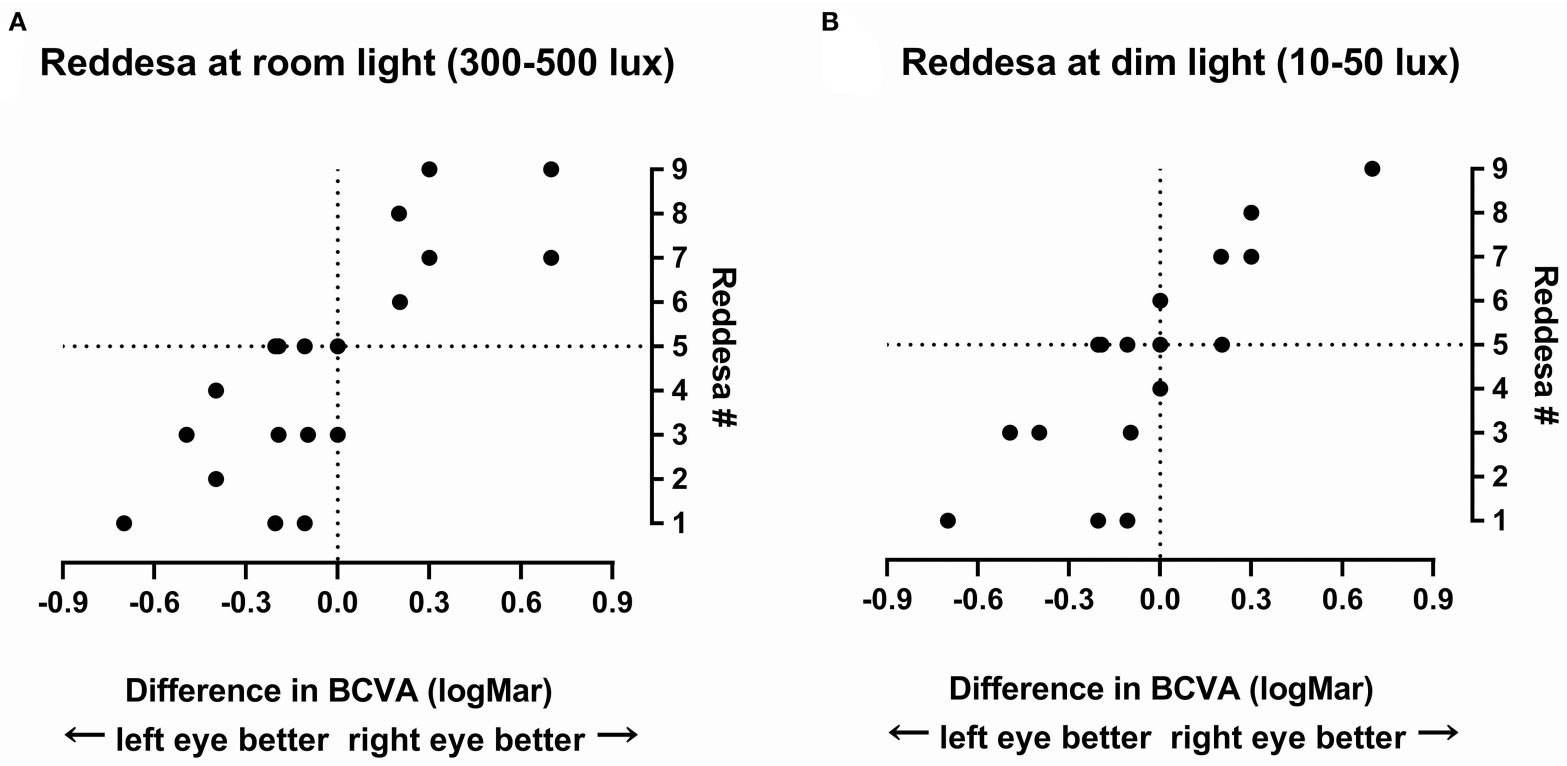
The result of the Reddesa test of each patient (*N* = 20) plotted against the difference of best-corrected visual acuity (BCVA; logMar) of the right and the left eyes in patients with unilateral optic neuritis at room light **(A)** and at dim light **(B)**.

It is difficult to grade the severity of the optic neuritis. To give an impression of the link between the results of the Reddesa chart and the severity of the optic neuritis, we plotted the difference in the BCVA (logMar) of the right and left eyes against the result of the Reddesa chart (Figure 2). A strong correlation was found between the BCVA difference and the Reddesa findings at room light (Spearman *r* = 0.79, *p* < 0.0001) and at dim light conditions (Spearman r = 0.81, *p* < 0.0001) in patients with optic neuritis.

A truly positive Reddesa test was found in 15 (75%) patients in the optic neuritis group at bright room light conditions (300–500 lux) and in 14 (70%) patients at dim light conditions (10–50 lux). If patients denoted a red desaturation in the Reddesa chart, it always indicated the correct side. No patient in the control group revealed a false positive Reddesa test under both light conditions.

The Reddesa test revealed a positive predictive value [true positives/(true positives + false positives) = 20/(20 + 0)] of 100% and a negative predictive value [true negatives/(true negatives + false negatives) = 20/(20 + 5)] of 80%.

## Discussion

The classic red desaturation assessment with the so-called red cap test is routinely performed in diagnosing optic neuritis (11). In our study, the majority of patients with optic neuritis (95%) described an alteration of the red color in the red cap test. However, the patients' description of the perceived difference varied widely and was often unspecific (pale, grayish, darker, opaque, minimal difference, or just different, etc.), making it sometimes difficult to identify the affected eye. As the result of the Reddesa test is a number, the results should be more comparable across patients, and the affected eye can easily be identified.

In addition, a low specifity of the red cap test has been reported (up to 25% wrong answers). This might lead to erroneously diagnose an optic neuritis in patients with no optic neuritis (10, 11). In our study, there were no wrong answers in the control group, both in the red cap test and in the Reddesa test. An explanation might be that our control group consisted of healthy subjects (mainly healthy hospital staff) with no expectations of any ophthalmological disease.

The Reddesa test allows a (semi-)quantification of the red desaturation, as patients have to decide which colors in both columns of the chart match the best. In our study, 15 out of 20 patients had a red desaturation in the Reddesa chart, but 5 patients had normal findings. However, in 2 of these 5 patients, there was no difference in the visual acuity and in another 2, the visual acuity was at least 1.0. This suggests a mild form of neuritis. Therefore, the Reddesa test–at least in this prototype version–might indeed miss milder forms of optic neuritis, and hence, it might be interesting to adapt the Reddesa scale for less severe cases.

The positive predictive value of the Reddesa test was good in this pilot study. In clinical routine, the majority of patients has no optic neuritis, and therefore, the negative predictivity value of the Reddesa test probably might be better as in this study.

All neuritis patients with a symptom duration of <3 weeks revealed an abnormal Reddesa test. In some patients with a long disease duration, the Reddesa test was normal. Therefore, it might be speculated that the reduced sensitivity in patients with longer disease duration of the test might be affected by recovery.

This study was a pilot study to analyze the feasibility of the Reddesa test in a clinical setting and has certain limitations. The patient number is low and the red cap was one (but not the only one) diagnostic criteria, leading to a potential bias. Although our trial showed the potential of the Reddesa test, a larger trial is needed to assess a potential superiority of the Reddesa test to conventional testing methods. It would then also be interesting to correlate the findings of the Reddesa test to visual evoked potentials (VEPs) and to correlate contrast sensitivity to different versions of our test, as some color tests correlate with contrast sensitivity (12).

We experienced the Reddesa test to be easily implemented into clinical routine as the testing time is short and the answers are clear. Furthermore, it allows the quantification of the red desaturation and therefore potentially also to monitor the severity and course of the disease. We believe that it can be easily performed by all kinds of medical personnel and not only by ophthalmologists. The test has a certain potential of becoming a quick and helpful tool for neurologists, pediatricians, and general practitioners in emergency rooms as also in private practices to triage affected patients. Based on this promising pilot study, further larger trials are desirable.

## Data Availability Statement

The original contributions presented in the study are included in the article. Further inquiries can be directed to the corresponding author.

## Ethics Statement

The study involving human participants was reviewed and approved by Kantonale Ethikkomission Bern. The patients/participants provided their written informed consent to participate in this study.

## Author Contributions

DB, A-LK, MA, and MD collected the data. MT, CT, A-LK, and DB analyzed and interpreted the data and wrote the first draft of the manuscript. MT designed the Reddesa chart. CT, MA, MT, and DG designed the study. All authors critically revised the manuscript and approved the final version.

## Conflict of Interest

Reddesa^®^ is a registered trademark by MT. The remaining authors declare that the research was conducted in the absence of any commercial or financial relationships that could be construed as a potential conflict of interest.

## Publisher's Note

All claims expressed in this article are solely those of the authors and do not necessarily represent those of their affiliated organizations, or those of the publisher, the editors and the reviewers. Any product that may be evaluated in this article, or claim that may be made by its manufacturer, is not guaranteed or endorsed by the publisher.
